# Cold Water in the Reduction of Hernia

**Published:** 1853-07

**Authors:** R. R. Gresham

**Affiliations:** Ebenezer, Miss.


### Art. III.—*Cold Water in the Reduction of Hernia* By R. R. Gresham, M. D., of Ebenezer, Miss.

The following case, in which cold water was successfully employed in the reduction of hernia, is deemed worthy of publication.

The case was one of oblique inguinal, or scrotal hernia, in a male servant. On the 5th of June, at 4 o'clock, P. M., the obstruction occurred. I was called to see him the next day about 5 o'clock, A M. When I arrived, I found the patient in the utmost pain and suffering. The hernial tumor was very large, and not disposed to yield to the usual remedies prescribed for the reduction of hernia. I gave him an anodyne, and left the following prescription:

℞ Wine Antimony
Tinct. Lobelia ää ℥ss.

M. Take 1ʒ every half hour. Apply cloths dipped in warm water every fifteen minutes; for the purpose of relaxing the muscular system. At 3 o'clock I saw the patient again, but found no amelioration of the symptoms; if anything, the sac was more tense, and the patient exhibited some incoherency of mind. I began to think I should have to operate, but concluded, before resorting to this last measure, to try the effect of an emetic and the application of cold water to the scrotum. The following prescription was given:

℞ Tart. emetic, gr. vi.
Tinct. Lobelia, ℥j.

M. Give ʒj. every fifteen minutes till free emesis occurs. This over, a gentle stream of cold water was let fall a distance of four or five feet on the tumor, while I administered, at intervals of half an hour, ʒss of the mixture already mentioned. The tumor began to recede under this treatment, and in the course of an hour and a half from the time the operation was commenced, the tumor was small enough to be grasped in my hand, and by gentle taxis returned to its proper place.

June, 1853.

